# *Mycobacterium tuberculosis* in a Trap: The Role of Neutrophil Extracellular Traps in Tuberculosis

**DOI:** 10.3390/ijms241411385

**Published:** 2023-07-13

**Authors:** Luiz Henrique Agra Cavalcante-Silva, Fernanda Silva Almeida, Arthur Gomes de Andrade, Fernando Cézar Comberlang, Leonardo Lima Cardoso, Shayenne Eduarda Ramos Vanderley, Tatjana S. L. Keesen

**Affiliations:** Immunology of Infectious Diseases Laboratory, Department of Cellular and Molecular Biology, Federal University of Paraiba, João Pessoa 58051-900, PB, Brazil; luiz0710@gmail.com (L.H.A.C.-S.); fernandaalmeida.ufpb@gmail.com (F.S.A.); arthurg.hit@gmail.com (A.G.d.A.); fcezar14@gmail.com (F.C.C.); leonardo15lima16@gmail.com (L.L.C.); shayenne.erv@gmail.com (S.E.R.V.)

**Keywords:** NETs, innate immunity, mycobacteria

## Abstract

*Mycobacterium tuberculosis* complex causes tuberculosis (TB), a disease that causes pulmonary inflammation but can also affect other tissues. Despite macrophages having a defined role in TB immunopathogenesis, other innate immune cells, such as neutrophils, are involved in this process. These cells have high phagocytic ability and a microbial-killing machine comprised of enzymes, antimicrobial peptides, and reactive oxygen species. In the last two decades, a new neutrophil immune response, the neutrophil extracellular traps (NETs), has been intensely researched. NETs comprise DNA associated with histones, enzymes, and antimicrobial peptides. These structures are related to antimicrobial immune response and some immuno-pathogenesis mechanisms. This mini review highlights the role of NETs in tuberculosis and how they can be helpful as a diagnostic tool and/or therapeutic target.

## 1. Introduction

Tuberculosis (TB) is an infectious-contagious disease mainly affecting the lungs and other tissues [1]. Several factors have limited TB control, including timely diagnosis and treatment, imperfect diagnostic testing, multi-drug resistance, and treatment abandonment [2,3,4,5]. Countries with a high burden of tuberculosis utilize Bacille Calmette-Guérin (BCG), a vaccine based on an attenuated strain of *Mycobacterium bovis*, to mitigate the risk of severe TB manifestations, such as meningitis and disseminated TB, in children. However, this vaccine has limited effects on adult pulmonary TB [6]. In 2020, approximately 10 million people had tuberculosis, but nearly 40% of these cases were undiagnosed and unreported [7]. This was followed by a rate of 1.5 million deaths from TB worldwide. The WHO’s End TB Strategy proposes targets of a 95% reduction in the absolute number of TB deaths and a 90% reduction in the TB incidence rate by 2035 [8].

The pathogenesis of TB is complex and involves many immune system cells, such as macrophages, lymphocytes, and neutrophils [9,10]. Among these, neutrophils may have a beneficial and a detrimental role in TB development [11]. The neutrophil’s functional repertoire encompasses the secretion of diverse antimicrobial peptides, as well as intricate communication with various immune cells. Additionally, neutrophils can release extracellular traps (NETs) (i.e., DNA, histones, and various granular and cytoplasmic proteins) that mediate many immune responses [12,13]. NETs play a crucial role in containing and eliminating bacteria by trapping and neutralizing them, preventing their spread to other tissues [14]. Moreover, NETs exhibit immunomodulatory functions, influencing the balance between pro-inflammatory and anti-inflammatory responses [15,16,17]. They can shape the immune environment, impacting disease progression, tissue damage, and the overall outcome of the infection [18]. Understanding the formation, regulation, and impact of NETs on the host immune response is essential due to their multifaceted involvement in TB pathogenesis. Thus, this mini review discusses the role of NETs in TB and how it can lead to the development of targeted interventions to enhance host defense mechanisms and improve treatment strategies for this global health burden.

## 2. Tuberculosis

Tuberculosis (TB) is one of the oldest infectious diseases in history [19]. It is caused by the *Mycobacterium tuberculosis* complex, comprising species including *M. bovis*, BCG strain of *M. bovis*, *M. africanum*, *M. caprae*, *M. microti*, *M. canettii*, and *M. pinnipedii*. Most TB cases are attributed to *M. tuberculosis (Mtb)*, which accounts for 98% of human cases [20,21].

Tuberculosis (TB) can manifest as either latent TB infection (LTBI) or active TB disease [22]. LTBI is asymptomatic and non-transmissible [23], while active TB disease presents with general symptoms such as fever, fatigue, weight loss, and respiratory symptoms, including persistent cough and coughing up blood in advanced cases [22]. A subset of individuals with active TB may be asymptomatic, termed subclinical tuberculosis. The active form develops in approximately 5 to 10% of individuals exposed to *Mycobacterium tuberculosis* (*Mtb*) [21,24,25,26]. In addition to pulmonary TB, extra-pulmonary TB (EPTB) is a significant clinical concern, as it can affect various body parts after the initial spread of *Mtb* through the pulmonary draining lymphatic system [27]. Symptomatic EPTB occurs in about 15% of infected individuals [28] and can involve various sites such as the peritoneum, kidneys, bone marrow, spleen, pleural, meningeal, peritoneal membranes, skin, and genito-urinary tract. Pulmonary TB is more prevalent due to the primary mode of *Mtb* transmission through the airways. EPTB can also manifest independently of pulmonary TB and can undergo both latent and active phases [24,29,30].

**Figure 1:**
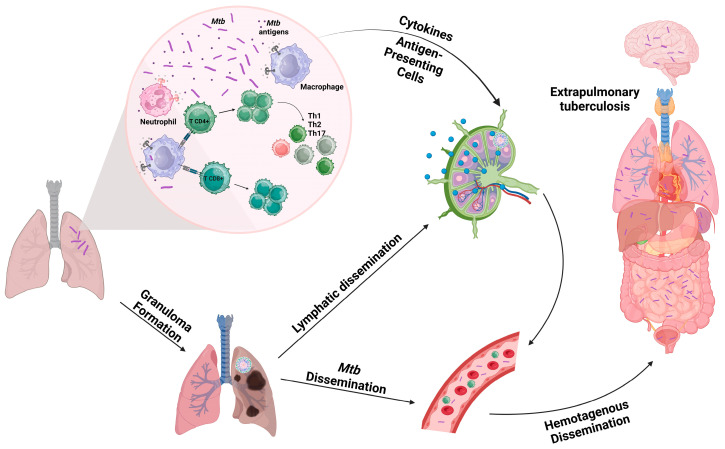
*Mycobacterium tuberculosis* (*Mtb*) infection begins with the recognition and internalization of bacteria by alveolar macrophages, allowing replication and establishment of infection. The immune response involves the secretion of cytokines and chemokines, recruitment of phagocytes, and activation of adaptive immunity, particularly CD4^+^ and CD8^+^ T cells. Monocytes and neutrophils are recruited to the infection site, potentially aiding bacterial dissemination. Dendritic cells play a crucial role in priming adaptive immune responses, presenting *Mtb* antigens to T cells in lymph nodes. In the lungs and other tissues, granulomas can form. They are a complex immune response mechanism aimed at containing and neutralizing pathogens, and their formation plays a significant role in the pathology of tuberculosis. Dissemination of *Mtb* from the lungs to extrapulmonary sites can occur through hematogenous spread or direct extension from adjacent lymph nodes or tissues. The immune response in extrapulmonary TB may exhibit variations depending on the involved tissue. Created with Biorender.com (accessed on 7 July 2023).

The pathogenesis of pulmonary and extrapulmonary tuberculosis involves intricate immunological mechanisms that shape the course and manifestations of the disease (For a comprehensive immunological review, please refer to [31]) (Figure 1). In pulmonary TB, *Mtb* is inhaled and reaches the alveoli of the lungs. Initial infection occurs when *Mtb* is phagocytosed by alveolar macrophages [32,33]. However, *Mtb* has evolved to evade the host immune response by resisting phagolysosomal fusion, allowing it to survive and replicate within macrophages [34]. This leads to the formation of granulomas, which are aggregates of immune cells, including macrophages, T cells, and dendritic cells. Within the granulomas, various immunological events take place [35]. Infected macrophages present *Mtb* antigens to CD4^+^ T cells, initiating a cell-mediated immune response. CD4^+^ T cells secrete many cytokines [36], particularly IFN-γ, which activates macrophages to enhance their antimicrobial activity. However, *Mtb* can also subvert the immune response by inhibiting phagolysosome maturation and suppressing pro-inflammatory cytokine production, thereby promoting its survival [34].

In most of the cases, the immune response successfully controls *Mtb* growth, leading to latent TB infection. However, if the immune system fails to control bacterial replication, active pulmonary TB develops [37]. In active disease, necrosis of infected macrophages occurs, leading to the release of *Mtb* and triggering further inflammation. This results in the formation of cavities within the lungs, which contribute to the spread of bacteria and transmission to others [38,39]. The epithelial cells, endothelial cells, and fibroblasts present at the initial site of infection are also susceptible to invasion. These cells possess the ability to elicit immune responses through the secretion of cytokines and other mediators [40].

Extrapulmonary TB typically occurs following the spread of *Mtb* from the initial pulmonary infection to the lymphatic system [27,41]. Furthermore, the dissemination of *Mtb* also takes place via hematogenous spread, whereby *Mtb* enters the bloodstream and can be transported to distant organs and tissues [42]. This dissemination allows *Mtb* to establish infection in different sites, leading to extrapulmonary manifestations. Similar to pulmonary TB, immune cells play a crucial role in the immunological response to extrapulmonary TB. In response to *Mtb* infection, cytokines and chemokines are secreted, attracting various immune cells to the site of infection [36]. This includes the recruitment of monocytes, neutrophils, dendritic cells (DCs), and CD4^+^ and CD8^+^ T lymphocytes [10,43]. Additionally, adipocytes, oligodendrocytes, and neurons have been observed to internalize *Mtb* and generate cytokines and chemokines [40].

The immunological mechanisms in extrapulmonary TB may differ depending on the affected organ or tissue and can be associated with immune dysregulation and impaired immune responses. Factors such as HIV infection, diabetes mellitus, and immunosuppressive therapies, can weaken the immune system and increase susceptibility to extrapulmonary TB [44]. Immune dysregulation can lead to uncontrolled bacterial replication, dissemination, and severe disease manifestations. 

The pathogenesis of both pulmonary and extrapulmonary TB involves intricate interactions among *Mtb*, host immune responses, and tissue-specific factors. A comprehensive understanding of the underlying mechanisms, including immune cells and their mechanisms, is crucial for the development of targeted interventions, improvement of diagnostic approaches, and optimization of treatment strategies for tuberculosis.

## 3. Neutrophils

Neutrophils comprise a significant portion of the circulating leukocytes in humans, accounting for approximately 50–70%. These cells have a characteristic segmented nucleus, are roughly 7–10 μm diameter, and play a critical role, especially in antimicrobial response [45,46,47,48,49,50]. The clinical significance of their abundance lies in predicting severe infections that may follow congenital or acquired conditions causing a decrease in neutrophil count in the bloodstream [51]. These cells were thought to be transcriptionally inactive. However, recent findings led to a paradigm shift, revealing subpopulation heterogeneity and crucial inflammatory and repair functions [52,53]. 

During development in the bone marrow, myeloid progenitors undergo sequential maturation to generate mature neutrophils that are released to the circulation in a process that gauges the extramedullary pool size. Although the kinetics of circulating neutrophils and their precursors remains somewhat uncertain, there has been increasing evidence about the remarkable plasticity that can be molded by the different tissues where they can be redistributed throughout neutrophil lifetime [54,55]. That can be explained by the transcriptional diversity of neutrophils during maturation, known as “neutrotime”, a transcriptional continuum for neutrophils in the bone marrow, blood, and spleen, suggesting a dominant developmental spectrum that underlies neutrophil heterogeneity under normal conditions [56]. Such diversity also includes low-density neutrophils (LDNs), which may manifest immunosuppressive or pro-inflammatory properties. Proinflammatory LDNs are known as low-density granulocytes (LDGs), and immunosuppressive LDNs are known as granulocytic myeloid-derived suppressor cells (G-MDSCs). Additionally, specific tumor-associated neutrophils (TANs) can be further categorized into N1 and N2 subsets, representing functions that are either anti-tumorigenic or pro-tumorigenic, respectively [57,58]. 

Neutrophil cytoplasm contains granules and secretory vesicles that play a crucial role in their function. As the first line of defense, neutrophils are the initial responders against a wide range of pathogens. These effector functions include phagocytosis with phagolysosome activity, degranulation of antimicrobial molecules and various enzymes involved in inflammation regulation, formation of neutrophil extracellular traps (NETs), and playing a role in tissue repair and wound healing by releasing growth factors [12,59,60].

The development of inflammation can be triggered by both infectious and sterile injuries, activating pro-inflammatory responses through pattern recognition receptors (PRRs) in response to pathogen-associated molecular patterns (PAMPs) and damage-associated molecular patterns (DAMPs) [61,62,63]. Neutrophils are recruited to inflamed tissues in both types of inflammation; however, their recruitment and subsequent pro-inflammatory responses may have detrimental effects in sterile injuries such as obesity-associated inflammation [64]. The ability of neutrophils to distinguish between infectious and sterile inflammation remains unclear since both types activate similar receptors and intracellular pathways. Different types of neutrophils, such as pro-inflammatory and pro-resolving, may be recruited depending on the specific ligands, cells involved, and type of inflammation [57].

Neutrophil recruitment involves upregulating adhesion molecules on endothelial cells, leading to tethering, rolling, and firm adhesion of neutrophils [12,65]. Chemokine receptors and adhesion molecules mediate the interaction between neutrophils and endothelial cells, followed by transmigration across the endothelial layer either paracellularly or transcellularly [66,67]. Neutrophils, traditionally considered pro-inflammatory cells, also exhibit anti-inflammatory and healing characteristics [64]. They aid in the removal of dead cells and bacteria, perform wound debridement, express proteases that benefit tissue repair, facilitate monocyte recruitment, and contribute to inflammation resolution, healing, and tissue repair. Additionally, neutrophils release growth factors that promote angiogenesis, which can be exploited by cancer cells to promote their growth and metastasis [68].

In TB immunopathogenesis, neutrophils have a dual role [11,69,70]. High levels of these cells can be found in patients with active TB in the bloodstream and bronchoalveolar fluid [71,72,73]. Additionally, these cells are involved in central nervous system tuberculosis development [74]. Furthermore, neutrophils can phagocyte mycobacteria, and their antimicrobial machinery can display some antimycobacterial activities [45]. Further, it has been demonstrated that alveolar mucosa increases the capacity of the neutrophil to recognize and kill *Mtb* [75]. However, the dysregulated granulocytic influx is related to disease progression [76], and massive neutrophilic infiltration has been found in mouse models of TB [77]. Thus, neutrophil is also implicated in the exacerbation of lung tissue damage. In the following section, we describe the role of neutrophil extracellular traps in tuberculosis.

## 4. Neutrophil Extracellular Trap in Tuberculosis

Neutrophil extracellular traps (NETs) are web-like structures composed of chromatin and antimicrobial proteins released by neutrophils. The primary function of NETs is to capture and kill microorganisms [78]. The DNA strands within NETs act as a physical barrier, immobilizing bacteria and fungi. At the same time, the antimicrobial proteins associated with the NETs, such as histones, neutrophil elastase, and myeloperoxidase, possess potent microbicidal properties that can directly kill pathogens [14]. Moreover, NETs also play a role in modulating immune responses. They can activate other immune cells, such as macrophages and dendritic cells, to enhance their antimicrobial activities. Additionally, NETs can interact with immune system components, including antibodies and complement proteins, facilitating the recognition and clearance of pathogens [79]. While NETs are crucial for host defense, their dysregulation has been associated with various inflammatory and autoimmune diseases. Excessive or prolonged NET formation can damage tissue, as NETs contain toxic components that can harm healthy cells and contribute to chronic inflammation [14,79,80,81,82].

The production of reactive oxygen species (ROS) and the initiation of protein kinase cascades are critical steps in the development and release of neutrophil extracellular traps (NETs) [83]. While not all the molecular mechanisms involved in the formation of these neutrophil traps have been fully elucidated, several central components have already been identified. Intracellular calcium ion (Ca^2+^) signaling is crucial for NET formation. Ca^2+^ acts as a second messenger and regulates several cellular processes involved in NETosis, including chromatin decondensation, granule release, and membrane disintegration [14,84]. The activation of the NADPH oxidase enzyme plays a pivotal role in NETosis by facilitating electron transfer to oxygen molecules, resulting in ROS formation [85]. Moreover, the stimulation of neutrophils with phorbol 12-myristate 13-acetate (PMA) is capable of inducing NET release through the activation of protein kinase C (PKC), a calcium-dependent proteins [14]. Building upon these discoveries, Hakkim et al. furthered our understanding of the molecular events involved in NET formation by uncovering its association with the Raf-MEK-ERK kinase pathway. According to their research, PKC participates in the formation of neutrophil traps by activating NADPH oxidase, which leads to ROS production. Additionally, the mitogen-activated protein kinase (MAPK) pathway positively regulates the expression of Mcl-1, an anti-apoptotic protein, thereby inhibiting apoptosis and facilitating cell death via NETosis [86]. 

Various stimuli, such as microbial products or inflammatory signals, can induce the translocation of peptidyl arginine deiminase 4 (PAD4) from the cytoplasm to the nucleus. PAD4 is an enzyme responsible for a post-translational modification called citrullination or deimination [87]. It catalyzes the conversion of peptidylarginine to peptidylcitrulline on histone proteins. PAD4-mediated histone citrullination is an essential step in NET formation. It leads to chromatin decondensation, allowing the DNA to become accessible for further processing and release [14]. Moreover, upon specific stimuli, such as inflammasome activation or microbial products, a protein called Gasdermin D (GSDMD) can be cleaved by caspase-1 or other proteases. This cleavage leads to the release of the N-terminal fragment of GSDMD, which forms pores in the plasma membrane. The pore-forming N-terminal fragment of GSDMD forms large openings, allowing the efflux of cytoplasmic contents, including NETs, into the extracellular space [88]. The rupture of the plasma membrane is a critical step in the release of fully formed NETs. Consequently, comprehending how these pathways can be targeted in tuberculosis holds great significance for therapeutic purposes.

Although NETs have infection control actions [89], the role of these neutrophil traps is still controversial and being investigated in diseases caused by mycobacteria [90]. Initially, it was only known that NETs could capture microorganisms. However, it is now recognized that such structures exert a direct microbicidal action against infectious agents [91]. Studies have already demonstrated the microbicidal activity of NETs against various pathogens, including bacteria, fungi, protozoa, and viruses [17,78,92,93,94]. However, other studies reveal that these extracellular structures only can capture but not kill *Mtb*, something already observed in vitro and in vivo, which may be related to the complex structure of its cell wall and its multiple detoxification mechanisms [95,96,97,98,99]. 

A current and relevant question regarding the role of NETs in tuberculosis is whether their release can be more beneficial or harmful to the host. This is because *Mtb* and BCG can mediate the release of NETs stimulation, and excessive formation of these structures can cause tissue damage [95,98,100]. Different mechanisms of ‘NETosis’ induced by *Mtb* and associated tissue damage have already been reported. Dang et al. [90] observed that the extracellular sphingomyelinase Rv0888 of *Mtb* in recombinant *M. smegmatis* could act as a virulence factor, inducing the release of NETs with associated myeloperoxidase (MPO), which leads to the aggravation of lung lesions through apoptosis via caspase-3. Additionally, extracellular traps induced by *Mtb* stimulate macrophages to release IL-6, TNF-α, and IL-1β, inflammatory cytokines that can recruit more neutrophils to the site of infection, exacerbating the inflammatory response and lung injury [101]. In addition, NETs associated with the NF-kB-dependent matrix metalloproteinase-8 (MMP-8) enzyme leading to collagen degradation in the lung tissue of humans with TB, and increased levels of NETs in patients’ sputum have been observed [102]. 

Teixeira et al. [103] also observed that NETosis could be induced by type 1 IFN through the blocking of GM-CSF, which leads to the exacerbated formation of NETs and consequent growth of mycobacteria and worsening of pulmonary pathology, reinforcing the evidence that neutrophil traps, when not controlled, contribute to the maintenance of pulmonary immunopathology in tuberculosis. Moreover, Su et al. [104] demonstrated that the induction of low-density granulocyte generation occurs during mycobacterium tuberculosis infection, facilitated by the promotion of neutrophil extracellular trap formation through the ROS pathway. The induction of ROS and neutrophil necrosis is triggered by products of the RD1 gene of the mycobacterium. *M. tuberculosis* mutants lacking gene products encoded by the RD1 region, such as ESAT-6, fail to induce necrosis in neutrophils and die, preventing the establishment of infection. Furthermore, patients with mutated NADPH oxidase genes resulting in defective ROS production and inhibited MPO action are protected from necrotic death, demonstrating an ROS-dependent death induced by *M. tuberculosis* [105,106].

Another mechanism capable of inducing NETosis is the release of ESAT-6 by the mycobacterial ESX-1 system [107]. Francis et al. [107] demonstrated that the ESAT-6 protein acts as a leukocidin, causing neutrophil necrosis through a high influx of Ca^2+^ that leads to NET release, correlating the ESX-1 system as one of the virulence factors of *Mtb*. Moreover, the release of these NETs not only removes the threat of neutrophils and creates a necrotic environment rich in nutrients and conducive to extracellular bacillary growth. Therefore, in addition to aggravating tissue damage, NETs, when released excessively, also contribute in other ways to the progression of the pathology, and *Mtb* seems to know how to exploit this well.

On the other hand, one of the characteristics of active pulmonary tuberculosis is the presence of necrotic cores containing leukocytes and extracellular bacteria, which can undergo cavitation. Therefore, these lesions are associated with the potential for airborne transmission of the disease [107,108]. In addition, these granulomas are hypoxic, leading to decreased formation of NETs, probably caused by the decrease of reactive oxygen species (ROS) [109]. However, the consequences of this decrease are still unclear.

Furthermore, studying markers that help indicate tuberculosis’s stages is paramount for its treatment. In this regard, Schechter et al. [110] observed that the release of NETs in TB could be identified through blood plasma by detecting extracellular DNA, human neutrophil elastase (HNE), and MPO. In addition, treatment with antibiotics decreased plasma levels of NETs. Similarly, De Melo et al. [111] reported higher levels of citrullinated histone H3, a marker of NETs, in the peripheral blood of patients with pulmonary tuberculosis and lung tissue damage. Additionally, Meier et al. [112] also observed increased levels of NETs in the total blood of tuberculosis progressors up to 6 months before the diagnosis of the disease, as well as their additional activation in tuberculosis patients. Such evidence suggests the detection of NETs could be an essential biomarkers in tuberculosis [73]. Furthermore, it again brings to light the formation of NETs associated with the immunopathology of the disease.

As previously mentioned, *Mtb* can induce the release of NETs but is not killed by these traps, which is interesting considering that NETs can lead to the death of other microorganisms. However, the mechanisms by which the mycobacterium evades extracellular traps have yet to be fully elucidated. Nevertheless, it is known that many bacterial species have developed ways to escape death by NETs. For example, *Streptococcus pyogenes*, *Staphylococcus aureus*, and *S. pneumoniae* produce DNases, which are seen as virulence factors for impairing NETosis [113,114,115]. Additionally, *S. pneumoniae* has a polysaccharide capsule that reduces the binding of NETs and modifies the lipoteichoic acids of its cell wall, altering its charge and disrupting its affinity with antimicrobial factors [115]. With the same goal, Group A *Streptococcus* (GAS) produces the M1 protein, inhibiting the action of the broad-spectrum antibacterial cathelicidin LL-37. Thus, inadequate LL-37 activity helps the survival of GAS within NETs [116]. Furthermore, it was demonstrated that LL37 complexed with DNA are internalized by macrophages after neutrophil release and then inhibit mycobacteria growth intracellularly [100]. Thus, evading this mechanism seems a plausible way for mycobacteria to escape NETs’ antimicrobial activity. 

Furthermore, bacteria can escape from NETs through other mechanisms, such as releasing peptidases, forming biofilm, and inhibiting ROS production [114]. Concerning *Mtb*, to what extent this mycobacterium exploits these escape mechanisms has yet to be fully clear. Still, it is known that mycobacteria have a complex cell wall structure with a high lipid content, which hinders the penetration of antibacterial agents [117]. Additionally, *Mtb* is also sensitive to the action of LL-37, and here, the inhibition of this peptide by the pathogen is also something that can be investigated. Likewise, its detoxifying mechanisms should be better studied to bring to light, in greater completeness, the factors that contribute to the survival of *Mtb* in NETs, which would aid in discovering new therapeutic targets for tuberculosis. 

Based on the molecular events involved in the formation of NETs and considering *M. tuberculosis* as a species that effectively induces NETosis for its own benefit, it is crucial to understand which molecules the mycobacteria employ to induce the formation of extracellular traps, disrupt host cell vesicular trafficking, interfere with leukocyte survival signaling pathways and impede innate immunity in various ways. Investigating possible inhibitors that target specific molecules discussed here, such as ESAT-6 and other gene products of RD1, could be highly valuable in inhibiting the pathogenic NETosis induced by the mycobacterium. Additionally, inhibiting protein kinases and enzymes involved in lipid biosynthetic pathways that enhance bacterial virulence may aid in the treatment of tuberculosis. Furthermore, the inflammatory response generated by the mycobacteria through cytokines such as IL-6, TNF-α, and IL-1β can also be targeted by immunotherapies that aim to counterbalance these pro-inflammatory cytokines, reducing the migration of neutrophils to necrotic sites that only facilitate cell-to-cell spread of *M. tuberculosis*. It is crucial to consider how NET-targeted therapies would interact with existing TB pharmacological treatments, such as antibiotics. Research could focus on evaluating the synergistic effects of combining traditional TB drugs with NET-modulating agents to determine if such combinations could improve treatment outcomes or reduce the duration of therapy. Therefore, further studies that effectively contribute to blocking these evasion mechanisms of *M. tuberculosis* and discovering new therapeutic targets are necessary.

## 5. Conclusions

**Figure 2:**
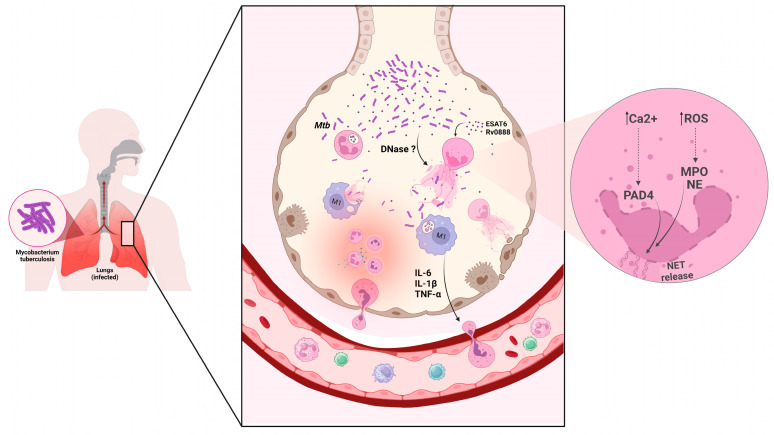
*Mycobacterium tuberculosis* (*Mtb*) and its antigens (e.g., ESAT-6 and Rv0888) induce neutrophil extracellular trap release (NETs). This mechanism can capture but not kill *Mtb*. The underlying mechanisms responsible for Mtb-induced release of neutrophil extracellular traps (NETs) involve the elevation of reactive oxygen species (ROS) levels and an increase in intracellular calcium ions (Ca^2+^). Experimental evidence has demonstrated that under other stimuli, ROS facilitates the activation of myeloperoxidase (MPO) and neutrophil elastase (NE), promoting their translocation from neutrophil granules to the nucleus, thereby triggering the subsequent release of NETs. Moreover, Ca^2+^ serves as a crucial signaling molecule, capable of activating numerous proteins, including peptidylarginine deiminase 4 (PAD4), an enzyme that plays a pivotal role in inducing histone citrullination—a critical step in the process of NET formation. Some resistance mechanisms may be associated (e.g., perhaps DNase release). The NETs can induce macrophages to release cytokines (e.g., IL-6, IL-1β, and TNF-α) that favor neutrophil influx. Macrophages can also internalize NETs; in macrophage lysosomes, NETs peptides could kill *M. tuberculosis*. Created with Biorender.com (accessed on 7 July 2023).

The role of neutrophil extracellular traps (NETs) in diseases caused by mycobacteria, particularly tuberculosis, is still controversial and under investigation. While studies have shown the microbicidal activity of NETs against various pathogens, including bacteria, fungi, protozoa, and viruses, some research suggests that NETs can capture but not kill *M. tuberculosis*, possibly due to the complex structure of its cell wall and evasion mechanisms. Additionally, excessive formation of NETs in TB can lead to tissue damage and exacerbate the inflammatory response and lung injury (Figure 2). However, NETs levels in the serum plasm of TB patients could be explored more to find a new way to detect the disease progression. Understanding the interaction between *M. tuberculosis* and NETs could lead to identifying new therapeutic targets for TB.

## Data Availability

Not applicable.
